# Pediatric Malignancies, Treatment Outcomes and Abandonment of Pediatric Cancer Treatment in Zambia

**DOI:** 10.1371/journal.pone.0089102

**Published:** 2014-02-21

**Authors:** Jeremy S. Slone, Catherine Chunda-Liyoka, Marta Perez, Nora Mutalima, Robert Newton, Chifumbe Chintu, Chipepo Kankasa, James Chipeta, Douglas C. Heimburger, Sten H. Vermund, Debra L. Friedman

**Affiliations:** 1 Division of Pediatric Hematology-Oncology, Department of Pediatrics, Vanderbilt University School of Medicine, Nashville, Tennessee, United States of America; 2 Department of Pediatrics and Child Health, University Teaching Hospital, and University of Zambia School of Medicine, Lusaka, Zambia; 3 Vanderbilt University School of Medicine, Nashville, Tennessee, United States of America; 4 Epidemiology and Cancer Statistics Group, University of York, York, England, United Kingdom; 5 Medical Research Council/Uganda Virus Research Institute Research Unit on Acquired Immune Deficiency Syndrome (AIDS), Entebbe, Uganda; 6 Division of General Internal Medicine and Public Health, Department of Medicine, Vanderbilt University School of Medicine, Nashville, Tennessee, United States of America; 7 Vanderbilt Institute for Global Health, Nashville, Tennessee, United States of America; 8 Division of Pediatric Infectious Diseases, Department of Pediatrics, Vanderbilt University School of Medicine, Nashville, Tennessee, United States of America; 9 Vanderbilt-Ingram Cancer Center; Nashville, Tennessee, United States of America; University of Massachusetts Medical School, United States of America

## Abstract

**Background:**

There exist significant challenges to the receipt of comprehensive oncologic treatment for children diagnosed with cancer in sub-Saharan Africa. To better define those challenges, we investigated treatment outcomes and risk factors for treatment abandonment in a cohort of children diagnosed with cancer at the University Teaching Hospital (UTH), the site of the only pediatric oncology ward in Zambia.

**Methods:**

Using an established database, a retrospective cohort study was conducted of children aged 0–15 years admitted to the pediatric oncology ward between July 2008 and June 2010 with suspected cancer. Diagnosis, mode of diagnosis, treatment outcome, and risk factors for abandonment of treatment were abstracted from this database and clinical medical records.

**Results:**

Among 162 children treated at the UTH during the study time period that met inclusion criteria, only 8.0% completed a treatment regimen with most of the patients dying during treatment or abandoning care. In multivariable analysis, shorter distance from home to the UTH was associated with a lower risk of treatment abandonment (Adjusted Odds Ratio [aOR] = 0.48 (95% confidence interval [CI] 0.23–0.97). Conversely maternal education less than secondary school was associated with increased risk for abandonment (aOR = 1.65; 95% CI 1.05–2.58).

**Conclusions:**

Despite availability of dedicated pediatric oncology treatment, treatment completion rates are poor, due in part to the logistical challenges faced by families, low educational status, and significant distance from the hospital. Alternative treatment delivery strategies are required to bring effective pediatric oncology care to the patients in need, as their ability to come to and remain at a central tertiary care facility for treatment is limited. We suggest that the extensive system now in place in most of sub-Saharan Africa that sustains life-long antiretroviral therapy for children with human immunodeficiency virus (HIV) infection be adapted for pediatric cancer treatment to improve outcome.

## Introduction

An estimated 12.7 million new cases of cancer occur each year worldwide, with 7.6 million deaths attributed to cancer. Fifty six percent of the cases and 64% of the deaths occur in low and middle income countries (LMIC) [1]. In children <15 years of age, 250,000 new cases of cancer are diagnosed annually but only about 20–30% of patients, mostly residing in high income countries, are adequately diagnosed and treated [2]. This significant gap in diagnosis and treatment exists despite the important advances in pediatric oncology that have produced dramatic improvements in survival rates in developed nations. In the United States, 80% of children with cancer survive [3]. However, in LMIC, notably those in sub-Saharan Africa, cure rates lag considerably, with often less than 25% of the children surviving [2], [4].

Many challenges exist in treating cancer effectively in LMIC including the lack of availability of common chemotherapeutic agents, cost of treatment, late stage at presentation, and limited radiotherapy and surgical resources making oncology care ultimately ineffective in achieving cure [5]. In addition, even when adequate oncologic treatments are available, disparities in education and socioeconomic conditions, coupled with inefficient or suboptimal health care delivery, result in poor outcomes for children diagnosed with cancer in LMIC.

The Republic of Zambia in sub-Saharan Africa faces many of these challenges. In 2010, the Vanderbilt University School of Medicine/Vanderbilt Institute for Global Health (VUSM/VIGH) and the University of Zambia School of Medicine/University Teaching Hospital (UNZA/UTH) formed a collaborative research relationship to evaluate the outcomes of cancer in Zambian children.

We sought to determine the most common cancers diagnosed at the country’s only pediatric cancer ward, profile the patients presenting for treatment of childhood cancer, and ascertain the outcome of cancer treatment. Furthermore, based on observations by the physicians at the UTH, abandonment of treatment was suspected to be a major cause of treatment failure. Therefore, we investigated the association of distance from home to the treatment center as well as other demographic, environmental, and disease-related factors with increased risk of abandonment of treatment.

## Methods

The study was approved by the Vanderbilt University Institutional Review Board (Nashville, TN, USA) and Ethics Reviews Converge (ERES) (Lusaka, Zambia). Requirements for consent were waived by the IRBs.

### Study Setting

**Figure 1:**
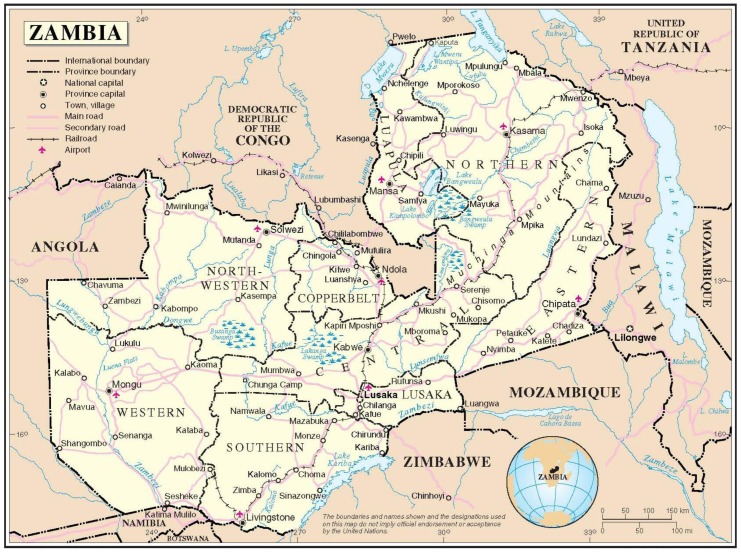
Zambia Map No.

The Republic of Zambia (Figure 1) is geographically the 39^th^ largest nation in the world, roughly the size of the US state of Texas [6]. Considered a LMIC by the World Bank, 59% of its population of 13 million people live in poverty and have an average life expectancy of 49 years [7]. The UTH is a 2000-bed tertiary care institution in the capital of Lusaka and serves as the country’s principal referral hospital and site of the only medical school in the country (UNZA) at the time of this study. UTH is currently the only government-funded institution in Zambia offering oncology care to both adults and children.

In the UTH’s Department of Paediatrics and Child Health, the Hematology-Oncology Unit has a 32 bed-capacity and offers chemotherapy. Radiation therapy became available with the establishment of the Cancer Disease Hospital (CDH) on the grounds of the UTH in 2006. However, there remain inadequate human resources to provide fully adequate services including a typical nurse to patient ratio of 1∶15. There is only one subspecialty trained pediatric hematologist-oncologist in Zambia, and there are limited opportunities for doctors or nurses to obtain further subspecialty training.

Basic diagnostic investigations such as complete blood count, liver and kidney function tests are readily available at the UTH at no cost to the patients. The UTH Department of Paediatrics and Child Health instituted routine opt out HIV testing in 2005 for every child admitted [8]. Diagnostic tests such as histopathology are available but the laboratory faces challenges such as frequent interrupted supply of reagents, inadequate staffing, and a demand for specimen processing that exceeds capacity. At the time of this study, immunohistochemistry, cytology, and molecular diagnostics were not available and imaging modalities were limited with one magnetic resonance imaging (MRI) and two computerized tomography (CT) imagers that serve the entire country, negatively impacting initial diagnosis or follow-up strategies.

Treatment with chemotherapy, radiation and surgery is offered at the UTH free of charge to citizens of Zambia. Chemotherapy protocols are derived from evidence-based protocols in the literature and are not necessarily adapted for LMIC. However, regardless of the protocol, inconsistent availability of cytotoxic drugs often dictates the regimen delivered to the patients, resulting in a lack of uniformity in treatment of specific cancers. A blood bank is available but demand exceeds available products due to limited resources to safely and efficiently distribute blood products. Many patients travel more than 500 km from their homes to receive care at UTH. Once treatment has commenced, patients and caregivers often must remain on the hospital grounds while awaiting the next cycle of chemotherapy due to lack of local housing and inability to return to home due to cost of travel.

### Study Population and Outcome Measures

**Figure 2:**
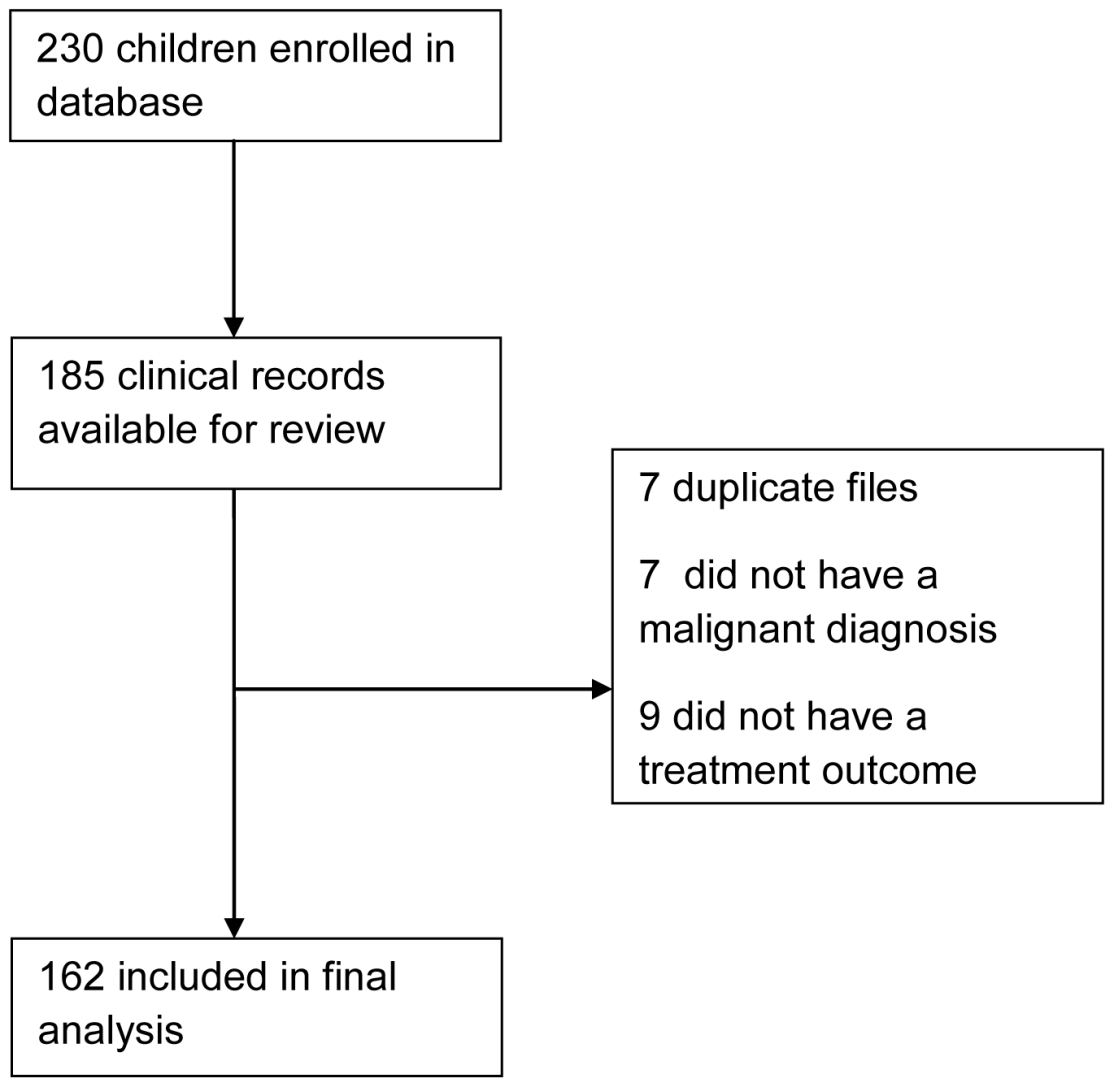
Consort Diagram.

A pre-existing database had been established at UTH in partnership with the University of York (UY) to investigate the etiologic linkage between viruses and malignancies, based on prior research by the same investigators in Malawi [9], [10]. All children aged 0–15 years who were admitted to the Paediatric Oncology Ward at the UTH between July 2008 and June 2010 with suspected cancer were enrolled in the UTH-UY database with caregiver consent and were tested for HIV. The database collected medical information like laboratory, pathology and radiology results as well as demographic and family information via an extensive face-to-face interview with the child’s caregiver. We constructed our retrospective cohort from this database then performed a chart review to verify malignant diagnoses and ascertain treatment outcomes (Figure 2).

Initial inclusion for the study was limited to patients registered in the database. Patients were subsequently excluded from the study if they did not have a malignant diagnosis confirmed by clinical or histopathological evaluation, were not residents of Zambia, or the clinical record could not be obtained to verify diagnosis and ascertain outcome. Clinical diagnoses were typically established based on history, physical exam, chest x-ray, ultrasound and occasionally CT scan.

Based on medical record review, one outcome assignment was determined for each patient: (1) completed treatment/actively undergoing therapy; (2) refused treatment; (3) abandoned treatment; or (4) death from any cause. Each child was classified with the first outcome that was met as determined by the medical record review. Abandonment of treatment was defined as the termination of care by the parent/caregiver and/or not presenting for scheduled treatment for>four weeks from the scheduled date of treatment. If a child returned to UTH after already having met the criteria for abandonment of treatment, his/her classification remained unchanged. Refusal of treatment was defined as no initiation of treatment after the diagnosis of a malignancy [11]–[13]. In compliance with the International Society of Paediatric Oncology (SIOP) Position Statement on abandonment of treatment, we combined refusal and abandonment of treatment for data analysis [14].

### Statistical Analyses

Data were collected from the UTH-UY database and the clinical charts by study personnel using a paper collection form. Data were later imported into a password-protected Microsoft Access database. Audits of >30% of the charts were performed to ensure data quality. Continuous variables were expressed as means and standard deviations. Categorical variables were expressed as percentages. Chi square tests or Fisher’s exact tests were used to evaluate statistical significance of associations between categorical variables, as appropriate. Student’s t-tests were used similarly to compare means for continuous variables. Kruskal-Wallis tests were utilized for continuous outcomes with more than two groups. Univariate and multivariable logistic regression were used to determine strength of association between risk factors and outcomes. To investigate abandonment of treatment, all treated patients were compared to the patients who had abandoned or had never initiated treatment. Treated patients included those who died during active treatment, completed treatment or were currently under treatment. Statistical analyses were done using STATA™, version 11 (StataCorp LP, College Station, TX, USA).

## Results

### Patient Characteristics

The database included 230 children admitted to the UTH Paediatric Oncology ward with a suspected malignancy during the enrollment period. Clinical records were recovered for 185 children. Seven medical records were duplicates, seven children did not have a malignant diagnosis, and nine did not have a treatment outcome ascertained, leaving 162 for the final data analysis. (Figure 2) Children presented to the UTH from throughout Zambia, with one third residing in Lusaka and Central provinces, closest in proximity to the UTH, and two thirds of the cohort residing in the other seven provinces at distances 300–800 km from the UTH. The mean ± standard deviation (SD) age at cancer diagnosis was 6.0±4.2 years. Of the participants included in the analyses, 55.6% were male. HIV serostatus was established in 159 of 162 (98%) cases; 10.5% were HIV-positive. (Table 1).

**Table 1:** Cohort Characteristics for 162 children with cancer seen at the University Teaching Hospital, Lusaka, Zambia*.

Cohort Characteristics	Completed Regimen/Active Treatment (N = 13, 8%)	Death From Any Cause (N = 75, 46.3%)	Abandonment of Treatment (N = 74, 45.7%)	Total	P value
*Age*, mean years (SD) (n = 162)	6.2 (3.6)	6.5 (4.2)	5.5 (4.2)	6.0 (4.2)	0.21
*Gender*, n (%) (n = 162)					
Female	6 (45.2%)	33 (44.0%)	32 (43.2%)	71 (43.8%)	0.88
Male	7 (53.8%)	42 (56.0%)	41 (55.4%)	90 (55.6%)	
Unknown	–	–	1 (1.4%)	1 (0.6%)	
*Mode of Diagnosis*, n (%) (n = 153)					
Clinical	6 (46.2%)	35 (50.7%)	33 (46.5%)	74 (48.4%)	0.87
Histopathological	7 (53.8%)	34 (49.3%)	38 (53.5%)	79 (51.6%)	
*Duration of Symptoms*, mean months (SD) (n = 154)	4.4 (5.1)	3.0 (4.1)	5.7 (6.1)	4.4 (5.3)	0.003
*HIV Status*, n (%) (n = 162)					
Positive	2 (15.4%)	11 (14.7%)	4 (5.4%)	17 (10.5%)	0.11
Negative	11 (84.6%)	61 (81.3%)	70 (94.6%)	142 (87.7%)	
Unknown	–	3 (4.0%)	–	3 (1.9%)	
*Father’s Employment Status*, n (%) (n = 87)					
Yes	3 (37.5%)	17 (40.5%)	8 (21.6%)	28	0.19
No	5 (62.5%)	25 (59.5%)	29 (78.4%)	59	
*Highest Maternal Education*, n (%) (n = 107)					
None	1 (12.5%)	1 (1.8%)	6 (13.6%)	8 (7.5%)	0.03
Lower Primary	–	2 (3.6%)	9 (20.5%)	11 (10.3%)	
Upper Primary	4 (50%)	29 (52.7%	20 (45.5%)	53 (49.5%)	
Secondary	3 (37.5%)	21 (38.2%)	9 (20.5%)	33 (30.8%)	
College	–	2 (3.6%)	–	2 (1.9%)	
*Malignancy,* n (%) (n = 162)					
Lymphomas	4 (30.8%)	20 (26.7%)	18 (24.3%)	42 (25.9%)	0.27
Wilms Tumor	1 ((7.7%)	16 (21.3%)	20 (27.0%)	37 (22.8%)	
Retinoblastoma	3 (23.1%)	12 (16.0%)	14 (18.9%)	29 (17.9%)	
Other Malignant Diagnosis	3 (23.1%)	4 (5.3%)	12 (16.2%)	19 (11.7%)	
Leukemias	–	7 (9.3%)	5 (6.8%)	12 (7.4%)	
Kaposi’s Sarcoma	1 (7.7%)	9 (12.0%)	2 (2.7%)	12 (7.4%)	
Neuroblastoma	–	4 (5.3%)	1 (1.4%)	5 (3.1%)	
Sarcoma (exc KS)	1(7.7%)	3 (4.0%)	2 (2.7%)	6 (3.7%)	
*Home Province* n (%), (n = 162)					
Lusaka	2 (15.4%)	15 (20.0%)	8 (10.8%)	25 (15.4%)	0.70
Central	3 (23.1%)	17 (22.7%)	9 (12.2%)	29 (17.9%)	
Copperbelt	1 (7.7%)	9 (12.0%)	8 (10.8%)	18 (11.1%)	
Southern	1 (7.7%)	5 (6.7%)	12 (16.2%)	18 (11.1%)	
Eastern	1 (7.7%)	6 (8.0%)	10 (13.5%)	17 (10.5%)	
North-Western	–	2 (2.7%)	1 (1.4%)	3 (1.9%)	
Western	–	5 (6.7%)	4 (5.4%)	9 (5.6%)	
Luapula	2 (15.4%)	6 (8.0%)	7 (9.5%)	15 (9.3%)	
Northern	3 (23.1%)	10 (13.3%)	15 (20.3%)	28 (17.3%)	

*For selected sociodemographic characteristics, sample size is <162 due to missing data. P-values calculated by Kruskal-Wallis test for continuous variables (age, time of symptoms) or Chi-square/Fisher’s exact tests (categorical variables) as appropriate. P-values for all categorical comparisons compare children whose cancer treatment was active at the time of the study or was completed vs. children died during therapy vs. abandoned treatment.

### Cancer Diagnoses

Only 51.6% of the cohort had a cancer diagnosis confirmed by histopathology, with the remainder diagnosed by clinical criteria. The most common diagnoses (clinical and/or histopathological) were lymphoma (25.9%), Wilms tumor (22.8%), and retinoblastoma (17.9%). Leukemia and Kaposi’s sarcoma accounted for 7.4% each. The mean time from the caregiver’s recognition of symptoms to presentation at the UTH was 4.4±5.3 months. (Table 1).

### Treatment Outcomes

Death from any cause during treatment (46.3%) and abandonment of treatment (45.7%), including nine refusals, were the most common outcomes; while only 8.0% completed a treatment regimen or were actively undergoing treatment, including palliative regimens, at the time of data collection. Length of symptoms, reported by the caretaker at initial enrollment into the UTH-UY database, prior to presentation at UTH, was associated with treatment outcome. Patients who died during therapy had the shortest duration of symptoms (mean 3 months, SD 4.1) while those that abandoned treatment had the longest duration of symptoms before presentation at the UTH (mean 5.7 months, SD 6.1, p = 0.003). (Table 1).

### Abandonment of Treatment

Factors associated significantly with abandonment of treatment included: lower risk if residing in Lusaka/Central provinces (Odds Ratio [OR] = 0.41 (95% confidence interval [CI]: 0.21–0.81; p = 0.01), higher risk with maternal education less than secondary school (OR = 2.73; 95%CI: 1.12–6.64; p = 0.03), and higher risk with negative HIV status (OR = 1.78; 95%CI: 0.99–3.19; p = 0.05). Mode of diagnosis, gender, access to running water, parental marital status, and paternal unemployment were not significantly associated with abandonment of treatment (Table 2). In a multivariable model including residence, HIV status, and maternal education, the associations with maternal education (Adjusted Odds Ratio (aOR) = 1.65; 95%CI: 1.05–2.58; p = 0.03) and Lusaka/Central province residence (aOR = 0.48; 95% CI 0.23–0.97; p = 0.04) retained statistical significance (Table 3).

**Table 2:** Factors Associated with Abandonment of Treatment (Unadjusted Analysis). Residence, HIV status and maternal education used in model.

Factors	Odds Ratio for Abandonment	95% CI	P
**Residence in Lusaka/Central Province**	0.41	0.21–0.81	0.01
**Histopathological Diagnosis**	0.87	0.46–1.64	0.66
**Male Gender**	0.91	0.50–1.67	0.76
**Access to Running Water**	0.96	0.43–2.11	0.93
**Married Parents**	0.90	0.37–2.16	0.81
**Mother’s Education <Secondary**	2.73	1.12–6.64	0.03
**Father Unemployed**	2.42	0.92–6.34	0.07
**Negative HIV**	1.78	0.99–3.19	0.05

**Table 3:** Factors associated with abandonment or non-initiation of treatment: Proximate residence, HIV status, and maternal education as predictors in a multivariable model.

Factors	Adjusted Odds Ratio for Abandonment	95% CI	P- value
**Residence in Lusaka/Central Province**	0.48	0.23–0.97	0.04
**Mother’s Education<Secondary**	1.65	1.05–2.58	0.03
**HIV Seronegative**	1.45	0.54–3.90	0.46

N = 162.

## Discussion

In the first review of pediatric oncology in Zambia in more than 15 years [15], we identified the demographic and disease characteristics of pediatric cancer patients presenting to the country’s only cancer treatment center, and report outcomes which were quite poor. Abandonment of treatment was highly prevalent and contributed to the poor outcomes and thus we explored risk factors. Notably, we found that the distance that parents have to travel with their children for ongoing cancer care is an evident impediment to successful adherence to pediatric oncologic services, as is limited maternal education.

An accurate assessment of the disease burden is essential in designing strategies to improve outcomes of pediatric cancer in LMIC. The epidemiology of childhood cancer is well documented in high-income countries, where population-based cancer registries exist, as contrasted with hospital-based registries or single institution analyses in LMIC, which may result in under-reporting [16]. According to Surveillance, Epidemiology, and End Results (SEER) age-adjusted incidence rates, there are 16 cases of cancer per 100,000 individuals less than 20 years old diagnosed each year in the United States [17]. Using this incidence and estimating that half of Zambia’s 13 million people are under the age of 20, we would expect at least 1000 cases of pediatric cancer per year in Zambia. However, in the two-year period of our study, only 230 patients <15 years old presented to the UTH with a suspected malignancy. Based on these assumptions, it is possible that only one of nine children with cancer in Zambia actually presented to the UTH for diagnosis and treatment during the study period. The remainder likely succumb to cancer without reaching the UTH due to lack of symptom recognition and awareness of need for urgent treatment, poor access to primary medical care, poor referral system, and the inability to travel to the UTH.

In addition to national cancer incidence, population-based cancer registries also provide vital information on the distribution of diagnoses in the studied population. According to the International Classification of Childhood Cancer, leukemia, (34%), brain tumors, (23%), and lymphoma (12%) represent the three most common diagnoses in children under the age of 15 years [18]. Different patterns of pediatric malignancy distributions have been reported in LMIC. Burkitt lymphoma, non-Hodgkin lymphoma, retinoblastoma, Wilms tumor and rhabdomyosarcoma are the most common childhood malignancies in Africa, compared to Asian countries, such as India and Pakistan, where leukemia is the most common malignancy [15], [19]–[25].

The distribution of malignancies that we identified in Zambia is consistent with reports from other LMIC in sub-Saharan Africa [26], but may represent selection bias due to a variety of factors. Three malignancies accounted for over 60% of the pediatric oncology diagnoses at the UTH (lymphoma, Wilms tumor and retinoblastoma). Due to limitations in pathology services, classification of lymphomas was mostly unavailable. The proportions of lymphoma and retinoblastoma have remained consistent since the last review of childhood cancer in Zambia, published by Chintu and colleagues in 1995 [15]. However, Wilms tumor now represents a much larger proportion of diagnoses than previously (22.3% vs. 3%) whereas Kaposi sarcoma (KS) is much less common in our cohort than in the prior report (7% vs. 19.5%) [15]. While this decrease in KS may be due the introduction of comprehensive effective HIV systems to prevent perinatal transmission [27], HIV-positive children and those with Kaposi sarcoma may be under-represented in our cohort, as their primary HIV care is in an outpatient setting. Inclusion in the UTH/UY database required an admission to the Paediatric Hematology-Oncology Ward at the UTH with a suspected malignancy. Leukemia represented only 7.4% of our cohort, much lower than seen in high income countries where it comprises more than 25% of diagnoses [28]. This may be due to the lack of recognition of symptoms of leukemia as compared with solid tumors, resulting in a lower rate of diagnosis. Prompt recognition, diagnosis and treatment of leukemia are paramount. However, the mean time of symptom to recognition by the child’s care giver to presentation at UTH in our cohort was over 4 months, likely contributing to children with leukemia dying in the absence of a diagnosis. The absence of brain tumors in our study population is due to a practice pattern in Zambia in which brain tumor patients typically are cared for by neurosurgeons and adult oncologists whose access to radiation therapy is more proximate to their clinics at the Cancer Diseases Hospital (CDH). We cannot compare our results with adult cancer programs in Zambia because integrated oncology did not exist until the CDH was established in 2006. Of note, Bowa et al noted a significant change in the pattern of adult malignancies presenting to UTH over the last 20 years with an increase in Kaposi’s sarcoma, cervical cancer and ocular cancer [29].

Establishment of a population-level tumor registry allows collection of accurate outcomes data. In Europe, overall 5-year survival for pediatric cancer is 81% [18]. In the United States, 80% of all children diagnosed with cancer survive [3]. Though longitudinal outcomes studies have not been performed in Zambia, our treatment completion rate of only 8% suggests that long-term survival of pediatric cancer in Zambia is drastically lower than seen in the US or Europe. Absence of effective national registries limits the ability to define a true pediatric cancer death rate.

The etiology of the significant cancer survival disparities between the US, Europe and LMIC is multifactorial. Provision of services for the prevention and treatment of cancer has not been a high priority for African governments due to the need to address more common and remediable causes of maternal and child mortality such as communicable diseases, HIV and malnutrition. In a review of pediatric oncology care in ten LMICs in 2006, management of pediatric cancer and access to care were poor or deficient in seven of the ten countries surveyed and accurate baseline data on incidence and outcome were very sparse. One key measure compared 5-year survival estimates for childhood cancer to health care spending per capita, and demonstrated that increased spending resulted in improved childhood cancer survival rates. Countries spending less than $20 US per capita on health care had the lowest survival at 5–10% (Bangladesh, the Philippines, Senegal, Tanzania and Vietnam). Survival rates increased to 30–60% when spending around $25–100 US per capita (Morocco, Egypt, Honduras, Ukraine, and Venezuela) [2]. According to the World Health Organization, Zambia spends $63 US per capita on health care [30]. This level of health care spending, based on the findings of the previously referenced study, should result in 30–60% 5-year survival of childhood cancer, but our study found that less than 10% of the children treated at the UTH even completed a treatment course. Thus, unlike other LMIC, the level of health care spending in Zambia does not appear to be positively associated with childhood cancer survival rates.

The major cause of treatment failure in our study was abandonment of treatment. The percentage of patients abandoning treatment in LMIC has been reported in other series to be around 25–50% [31]–[39]. In our cohort, 45% of the patients abandoned treatment. Previous studies investigating risk factors for abandonment of treatment have identified risk factors common across continents and regions. Reasons for abandonment are complex, but often include financial constraints and lack of parental education [31], [34], which are consistent with our finding that children were more likely to abandon treatment if the mother had limited education [36]–[38]. Social and economic constraints in Nigeria were identified as a hindrance to treatment of children with Burkitt lymphoma; only about 51% of children remained on treatment after 6 months of follow up [34]. In Malawi, family interviews revealed that absence from home and extra costs during the stay in the hospital were important concerns for parents [40]. Similar findings were noted in a study in Indonesia [39]. In Guatemala, 38% of children abandoned therapy within 6 months; adherence was associated with the presence of electricity, a television, or radio in the home and more than three rooms in the house. Fewer than 3 years of paternal elementary school education correlated with abandonment [41]. In India, parents of children who abandoned were found to have limited education and economic means [42].

In our cohort, we observed that closer proximity to the treatment center from home was associated with decreased risk of treatment abandonment. Two-thirds of our cohort resided in provinces over 300 km from the UTH located in a major urban center. A recent study of retinoblastoma patients in India found that abandonment of treatment was more common in patients from rural areas [43].

While distance to the treatment center was found to be a risk factor for abandonment of care in our study, its affect may be subject to many factors including adequacy of roads, availability of public transport and the amount of time required to travel to the treatment center. Bonilla and colleagues found that in El Salvador parental illiteracy and low monthly household income were associated with abandonment of treatment but not distance to the treatment center. However, the median travel time to the treatment center in the Salvadoran study was only 2 hours (range 1–3 hours) [11]. Distance to UTH may have also played a role in delaying initial presentation of the children who would eventually abandoned treatment in our study, as their mean duration of symptoms was the longest of the three outcomes groups (5.7 months vs. 3 months for the patients who died and 4.1 months for those who completed therapy). Further study is needed to elucidate factors preventing timely presentation to the UTH, as they may overlap with the barriers that predispose to abandonment of treatment. Our results suggest that to improve pediatric cancer outcomes in Zambia, the Zambian health care system may wish to consider ways to provide treatment closer to where the children reside or to provide temporary housing and other social supports at the UTH during cancer treatment.

Studies have shown that addressing socioeconomic barriers can retain children in treatment. By providing lodging, food, and transportation assistance to patients with acute lymphoblastic leukemia (ALL) in Brazil, abandonment of treatment was nearly eliminated over a 20 year period [44]. In Indonesia, a parental education program for ALL patients was introduced to increase the access to information about leukemia. After introduction of the program, treatment refusal decreased from 14 to 2%, and survival improved [36]. In El Salvador, with extensive financial and social support, only 13% of ALL patients abandoned care [11]. SIOP released a position statement in 2011 calling for more uniform studies on the cause of abandonment of treatment to design effective interventions [14].

Strengths of our study included the existence of a comprehensive database providing extensive demographic data on our subjects and the availability of most medical records. Although our data provide the most extensive review of the presentation and treatment outcomes of pediatric cancer in Zambia, and we have identified several areas prime for an intervention to decrease abandonment of care and improve outcomes, our study has several limitations. Histopathological diagnosis was not available for half of our patients, which may have led to misclassification of diagnoses. Twenty percent of potentially eligible participants in the cohort were excluded because their clinical records could not be located; this may have led to some bias. Due to the retrospective nature of our study, we were not able to determine cause of death or if cancer therapy was with curative or palliative intent. There was no method for longitudinal follow-up of survivors or those who abandoned treatment.

## Conclusion

Zambia is a striking example of the challenge of treating pediatric cancer in a LMIC. Barriers include access to primary care, diagnostic services, and effective treatment. Our study found that less than 10% of children presenting to the only government-funded pediatric cancer center actually completed treatment. As in many LMIC, abandonment of treatment was a significant cause of treatment failure. Strategies must be developed to assist families so their children may complete all cancer care, and may include enhanced parental education and provision of temporary housing during treatment.

A model for advances in pediatric health care in sub-Saharan Africa exists with the successful implementation of health care services for children with HIV. However, despite significant investment into the care of children with HIV, barriers continue to prevent optimal care especially to children from rural areas [45]. Therefore, the challenges to proper diagnosis and treatment of pediatric cancer in Zambia are representative of barriers throughout the medical system. Characteristics of successful HIV care have been identified to optimize the diagnosis and treatment of HIV across sub-Saharan Africa [46]. A major opportunity may exist to forge a strategic partnership with the now vast HIV care and treatment services for children in many LMIC, especially in sub-Saharan Africa [47]. Joining forces with HIV initiatives could enable clinicians to provide accessible curative or palliative care to children with cancer in such a way that fosters adherence to treatment and decreases abandonment of treatment.
